# Demographics, Radiological Findings, and Clinical Outcomes of *Klebsiella pneumonia* vs. Non-*Klebsiella pneumoniae* Pyogenic Liver Abscess: A Systematic Review and Meta-Analysis with Trial Sequential Analysis

**DOI:** 10.3390/pathogens11090976

**Published:** 2022-08-26

**Authors:** Kai Siang Chan, Christopher Tze Wei Chia, Vishal G. Shelat

**Affiliations:** 1 Department of General Surgery, Tan Tock Seng Hospital, 11 Jalan Tan Tock Seng, Singapore 308433, Singapore; 2 Department of Gastroenterology, Tan Tock Seng Hospital, 11 Jalan Tan Tock Seng, Singapore 308433, Singapore; 3 Lee Kong Chian School of Medicine, Nanyang Technological University, 11 Mandalay Rd., Singapore 308232, Singapore; 4 Yong Loo Lin School of Medicine, National University of Singapore, 10 Medical Dr., Singapore 117597, Singapore

**Keywords:** *Escherichia coli*, hepatic abscess, liver abscess, *Klebsiella pneumoniae*, percutaneous drainage, surgical drainage

## Abstract

Pyogenic liver abscess (PLA) is a common cause of hepatobiliary sepsis. *Klebsiella pneumoniae* (KP) is the most common organism causing PLA. Evidence is scarce on the demographics, radiological findings, and outcomes of KPPLA versus non-KPPLA (N-KPPLA). PubMed, Embase, The Cochrane Library, and Scopus were systematically searched until 14 May 2022 for studies comparing KPPLA and N-KPPLA. Exclusion criteria were single-arm studies. Primary outcomes were mortality (30-day/in-hospital) and metastatic complications. There were 16 studies, including 5127 patients (KPPLA n = 3305, N-KPPLA n = 1822). Patients with KPPLA were younger (mean difference: −2.04 years, *p* = 0.02). History of hepatobiliary disease (Odds ratio (OR) 0.30, 95% CI: 0.20, 0.46) and malignancy (OR 0.26, 95% CI: 0.16, 0.42) were less common in KPPLA. KPPLA was associated with lower incidence of multiple abscesses (OR 0.52, 95% CI: 0.35, 0.76, *p* < 0.001) and bilobar abscesses (OR 0.60, 95% CI: 0.49, 0.74, *p* < 0.001). KPPLA has higher overall metastatic complications (KPPLA 9.7% vs. N-KPPLA 4.8%, OR 3.16, 95% CI: 2.00, 4.99, *p* < 0.001), but lower mortality (KPPLA 3.9% vs. N-KPPLA 7.6%, OR 0.51, 95% CI: 0.34, 0.78, *p* < 0.001). Trial sequential analysis showed conclusive evidence that KPPLA has lower mortality than N-KPPLA. In conclusion, KPPLA has lower mortality than N-KPPLA.

## 1. Introduction

Pyogenic liver abscess (PLA) is a common global public health problem as it contributes to 13% of intra-abdominal abscesses [1]. With advancements in diagnostic microbiology, imaging technology, improved understanding of sepsis and critical care, and minimally invasive image-guided interventions such as percutaneous drainage (PD), clinical outcomes continue to improve; however, PLA-related mortality remains high, in the range of 10–30% [2]. Patient factors, disease factors, and treatment-related factors determine the clinical outcomes of PLA. Patient factors include underlying hypertension, diabetes mellitus (DM), Eastern Cooperative Oncology Group (ECOG) performance status, and Acute Physiology and Chronic Health Evaluation (APACHE) II score [3,4,5]. Disease factors include size, number of abscesses, and presence of gas formation [6,7]. Treatment-related factors include policies for PD, adoption of a liver abscess care bundle, and compliance with the sepsis bundle with local antimicrobial stewardship initiatives [8,9].

Microbiology of PLA has also been shown to impact outcomes in PLA [8]. *Klebsiella pneumoniae* (KP) is the leading causative organism for PLA, followed by *Escherichia coli* (EC) [2,10,11]. *Klebsiella pneumoniae* pyogenic liver abscess (KPPLA) is associated with DM and gas formation, possibly impacting clinical outcomes [7,12]. Some studies have also reported that KPPLA is associated with solitary abscess allowing for easier PD [13], but this has been refuted by others [14]. Serious metastatic complications secondary to haematogenous seeding (e.g., endogenous endophthalmitis) have also been reported with non-hypervirulent and hypervirulent strains, with a reported incidence of 3–11% [15]. ECPLA is more common in older patients and patients with underlying cardiac co-morbidity and biliary disease, and has been associated with small and multiple abscesses, which could impact clinical outcomes [8,16]. PLA due to other aetiologic organisms are uncommon and are not widely compared with KPPLA. To date, there are isolated reports of outcomes of KPPLA or non-KPPLA (N-KPPLA) from various institutes. However, only a few studies directly compare KPPLA with N-KPPLA [8,16]. To our knowledge, there has been no review comparing these groups. Hence, our study aims to bridge the knowledge gap by summarizing the existing literature on the clinical demographics, radiological findings, and clinical outcomes of KPPLA vs. N-KPPLA.

## 2. Results

**Figure 1:**
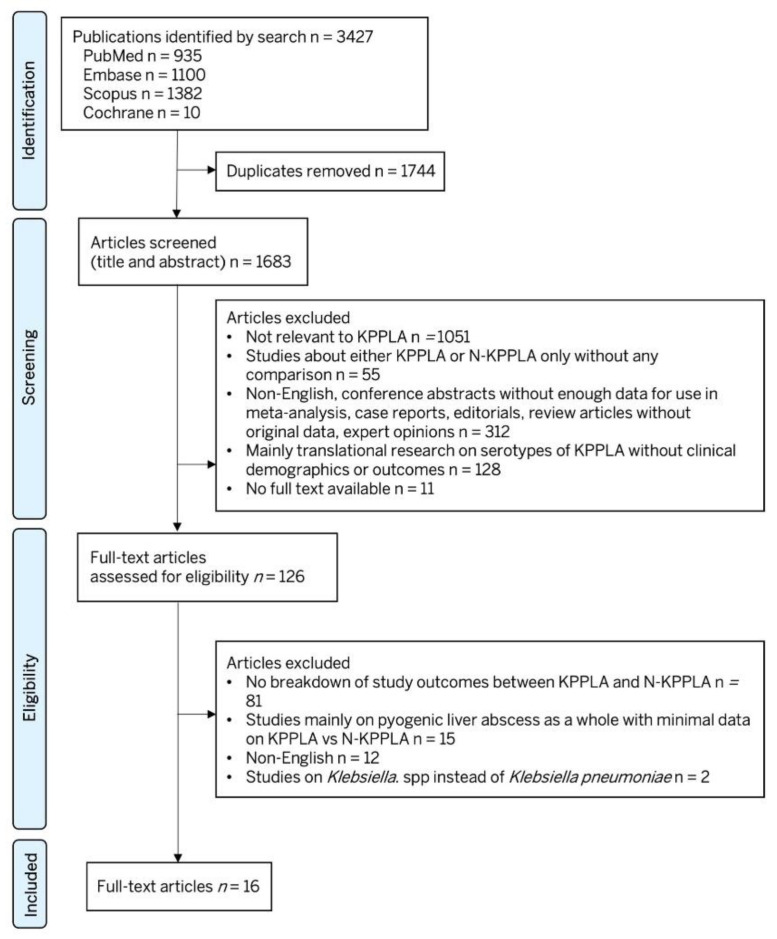
PRISMA (Preferred Reporting Items for Systematic reviews and Meta-Analyses) flowchart for study selection.

Our systematic search identified 3427 articles from four databases, with 1683 articles remaining after removal of duplicates. Titles and abstracts of all the identified articles were screened. Subsequently, 179 full-text articles were reviewed for inclusion in the study. A total of 16 articles were included in the final analysis. Figure 1 shows the PRISMA diagram for the study selection process. Funnel plots are appended in Supplementary Figure S1. None of the study variables or outcomes had significant publication bias using Egger’s regression test, except for in the incidence of multi-loculated abscess (*p* = 0.007) and bilobar abscess (*p* = 0.028).

### 2.1. Study Characteristics and Patient Demographics

The overall study characteristics and patient demographics are summarized in Table 1.

A total of 16 studies including 5127 patients (KPPLA n = 3305, N-KPPLA n = 1822) were included [8,13,14,16,17,18,19,20,21,22,23,24,25,26,27,28]. Most of the studies were conducted in East Asia (n = 15/16). There were 64.3% males (overall n = 2821/4390, KPPLA n = 1744/2634 (66.2%), N-KPPLA n = 1077/1756 (61.3%)). Patients who had KPPLA were more likely to be younger (MD: −2.04 years, 95% CI: −3.69, −0.38, *p* = 0.02) (Supplementary Figure S2A), males (OR 1.35, 95% CI: 1.03, 1.77, *p* = 0.03) (Supplementary Figure S2B), and have DM (KPPLA n = 1605/3210 (50.0%), N-KPPLA n = 524/1786 (29.3%), OR 2.35, 95% CI: 1.76, 3.14, *p* < 0.01) (Supplementary Figure S2C). History of hepatobiliary disease (OR 0.30, 95% CI: 0.20, 0.46, *p* < 0.01) (Supplementary Figure S2D) and underlying malignancy (OR 0.26, 95% CI: 0.16, 0.42, *p* < 0.01) (Supplementary Figure S2E) were less common in KPPLA compared to N-KPPLA. 

### 2.2. Biochemistry Findings

There were 6 studies with 810 patients (KPPLA n = 542, N-KPPLA n = 268) which reported on total white cell (TWC) count [13,18,21,22,25,26]; there was no difference in TWC between KPPLA and N-KPPLA (MD 0.28, 95% CI: −0.37, 0.93, *p* = 0.39).

### 2.3. Radiological Findings

There were 13 studies that reported on radiological findings overall (US n = 4, CT n = 6, either the US and/or CT n = 3) [8,13,14,16,17,18,19,20,21,22,24,25,26]; three studies reported the use of US and/or CT for imaging but did not specify whether the radiological findings were from US or CT. Supplementary Figure S3 illustrates the forest plots of the radiological findings of KPPLA versus N-KPPLA. KPPLA was associated with lower incidence of multiple abscesses (OR 0.52, 95% CI: 0.35, 0.76, *p* < 0.001) (Supplementary Figure S3A) and bilobar abscesses (OR 0.60, 95% CI: 0.49, 0.74, *p* < 0.001) (Supplementary Figure S3B), but was associated with higher incidence of multi-loculated abscesses (OR 3.19, 95% CI: 1.45, 7.00, *p* < 0.001) (Supplementary Figure S3C).

Gas formation (OR 1.04, 95% CI: 0.59, 1.83, *p* = 0.90) (Supplementary Figure S3D), size of PLA (MD −0.08 cm, 95% CI: −0.71, 0.55, *p* = 0.80) (Supplementary Figure S3E), and presence of a solid component of PLA (OR 7.05, 95% CI: 0.87, 57.21, *p* = 0.07) (Supplementary Figure S3F) were comparable between KPPLA and N-KPPLA. Table 2 summarizes the radiological findings and clinical outcomes between KPPLA and N-KPPLA.

### 2.4. Clinical Outcomes 

Patients with KPPLA were less often managed with conservative therapy alone (OR 0.49, 95% CI: 0.32, 0.74, *p* < 0.001), and were more likely to undergo PD (OR 1.48, 95% CI: 1.09, 2.02, *p* = 0.01). Incidence of percutaneous aspiration was comparable between KPPLA and N-KPPLA (OR 1.32, 95% CI: 0.74, 2.37, *p* = 0.35). There was a trend towards lower SD (OR 0.69, 95% CI: 0.44, 1.08, *p* = 0.11) and shorter LOS (MD: −3.93 days, 95% CI: −7.98, 0.13, *p* = 0.0577) (Supplementary Figure S4A) in KPPLA compared to N-KPPLA but this did not achieve statistical significance.

There were 8 studies with 1780 patients (KPPLA n = 1304, N-KPPLA n = 476) which reported on overall metastatic complications [13,14,16,19,21,25,26,27]. KPPLA was associated with higher overall metastatic complications (KPPLA n = 126/1304 (9.7%), N-KPPLA n = 23/476 (4.8%), OR 3.16, 95% CI: 2.00, 4.99, *p* < 0.001) (Figure S4B). There was a trend towards higher endophthalmitis (KPPLA n = 20/1162 (1.7%) vs. N-KPPLA n = 1/297 (0.3%), OR 2.80, 95% CI: 0.84, 9.34, *p* = 0.09) (Figure S4C) and brain abscess/meningitis (KPPLA n = 10/397 (2.5%), N-KPPLA n = 0/267, OR 3.22, 95% CI: 0.68, 15.19, *p* = 0.14) (Figure S4D) compared to N-KPPLA, though this did not reach statistical significance. There was one study that reported on thrombophlebitis of hepatic veins and/or tributaries [13]; they demonstrated a higher incidence of thrombophlebitis in KPPLA compared to N-KPPLA (KPPLA: n = 28/92 (30.4%) vs. N-KPPLA: n = 2/39 (5.1%), *p* < 0.01). There were 11 studies with 2375 patients (KPPLA n = 1833, N-KPPLA n = 542) which reported on mortality [8,13,14,16,17,21,24,25,26,27,28]. Mortality was lower in KPPLA (KPPLA 3.9% vs. N-KPPLA 7.6%, OR 0.51, 95% CI: 0.34, 0.78, *p* < 0.001) (Figure S4E).

**Figure 2:**
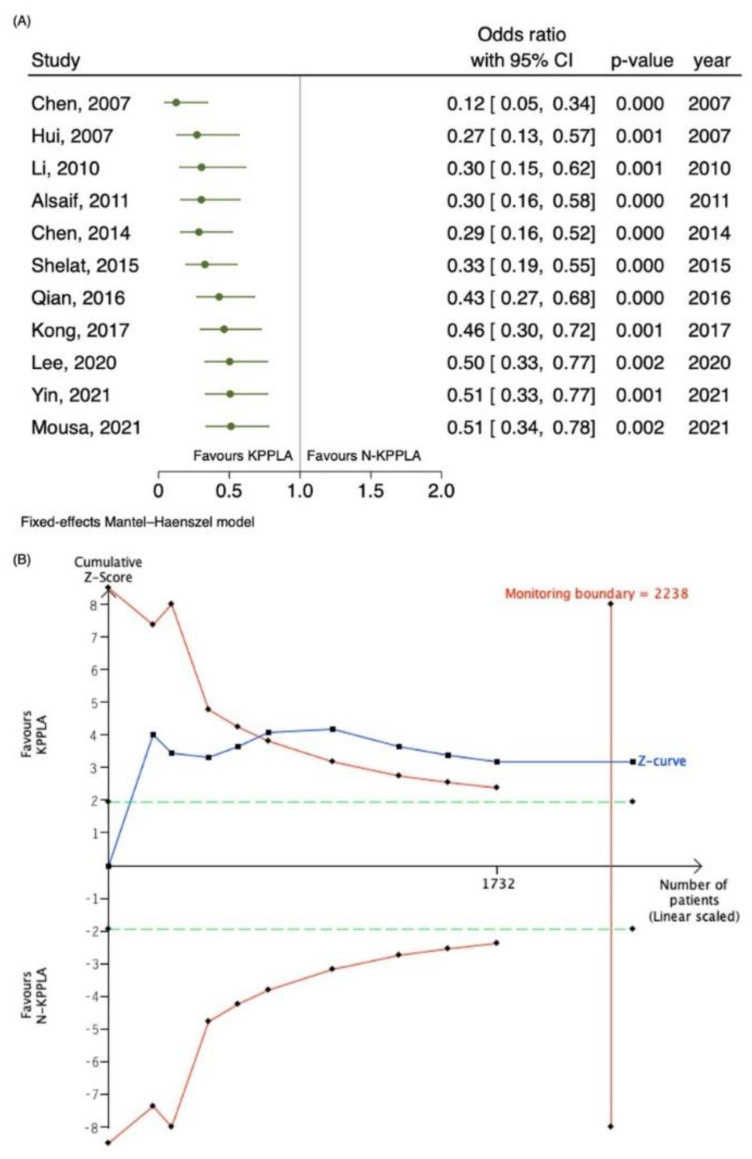
(**A**) Cumulative analysis by year on the mortality of KPPLA versus N-KPPLA [8,13,14,16,17,21,24,25,26,27,28] (**B**) Trial sequential analysis regarding mortality. The cumulative z-curve (blue line) was constructed using a fixed-effects model which crossed both the conventional boundary (dotted green line) (z score = 1.96, two-sided *p*-value = 0.05) and the trial sequential monitoring boundary (TSMB) (red line) for benefit, establishing sufficient and firm evidence that further trials are not required. Diversi-ty-adjusted requirement information size of 2238 was calculated using α = 0.05 (two-sided) and a power of 80%. Two studies (Mousa 2021 and Yin 2021) [14,28] were excluded from the calculation of the TSMB in view of low incidence of events (resulting n = 1732 patients).

Figure 2A shows the cumulative analysis of mortality reported by studies temporally. First published results in the late 2000s showed statistically significant effects. Studies which were added later resulted in a steady decrease in effect, with the association between KPPLA and lower mortality remaining statistically significant. The addition of the latest studies by Yin et al. and Mousa et al. made little change to the point estimate and may signal a plateau in evidence [14,28]. Trial sequential analysis (TSA) showed that the cumulative Z-curve exceeded the trial sequential monitoring boundary (TSMB) in favour of KPPLA for lower mortality and crossed the required information size (Figure 2B). This suggests firm evidence that mortality in KPPLA is conclusive without need for further studies to investigate mortality between KPPLA and N-KPPLA.

TSA showed that the cumulative patient numbers exceeded the required information size of 2310, and the cumulative Z-score exceeded the significant boundary in favor of KPPLA for lower mortality (Figure 2B), suggesting that the meta-analysis was conclusive. No further studies are required to investigate mortality between KPPLA and N-KPPLA.

## 3. Discussion

This is the first systematic review and meta-analysis comparing patient demographics, radiological findings, and clinical outcomes between KPPLA and non-KPPLA. This study showed that KPPLA is associated with younger patients and a higher incidence of DM. On the other hand, N-KPPLA is associated with a higher incidence of history of hepatobiliary disease and malignancy. KPPLA are more commonly solitary and unilobar but are associated with multi-loculation. KPPLA has a higher incidence of overall metastatic complications but has lower overall mortality compared to N-KPPLA.

KP is the most common pathogen widely and increasingly implicated in the causation of PLA [29]. Due to the unique peculiarities of KP, it is possible that clinical outcomes of PLA could differ based on microbial aetiology. For example, KPPLA is associated with metastatic complications, coined as KP invasive liver abscess syndrome (KPIS). KPIS was first reported in the 1980s in Southeast Asia [30]. Our study validates this finding, with overall higher metastatic complications in KPPLA than N-KPPLA (OR 2.95, 95% CI: 1.80, 4.84, *p* < 0.001). This phenomenon has been attributed to the unique virulent characteristics of KP (coined as hypervirulent KP (hvKP)), which includes hypermucoviscosity phenotype in K1 and K2 capsular serotypes, mucoviscosity-associated gene A (magA), and regulator of mucoid phenotype A (rmpA) [31]. Wang et al. showed a high prevalence of hvKP (89.1%) in their study on 131 KP isolates, with increased expression of rmpA and aerobactin [18]. Aerobactin allows KP to compete for iron sources in the host and plays an important role in the virulence of KP [32]. Endophthalmitis, one of the metastatic complications of KPPLA, has also been reported to be increasingly prevalent as a cause of endogenous endophthalmitis in Asia, ranging from 54–61% [15,33,34]. While our meta-analysis failed to demonstrate a significant difference in endophthalmitis between KPPLA and N-KPPLA (OR 2.80, 95% CI: 0.84, 9.34, *p* = 0.09), this is likely attributed to the small number of studies (n = 5) and low incidence of endophthalmitis (KPPLA n = 20/1162 (1.7%) vs. N-KPPLA n = 1/297 (0.3%)). Similarly, we failed to show higher incidence of brain abscess or meningitis in KPPLA; but caution should be taken to interpret these results due to the low sample size and positive cases reported. 

DM has been reported to be a risk factor for KPPLA [35,36]. Our meta-analysis similarly demonstrated a higher incidence of DM in patients with KPPLA with pooled OR of 2.35. While higher metastatic complications of KPPLA have been attributed to hvKP, it has been postulated that DM is a host susceptibility factor for metastatic complications [37]. Translational research by Lee et al. showed that DM is associated with a slight reduction in Th1 cytokine production in mononuclear cells, which may impact efficient control and clearance of infection [38]. DM also increases the expression of G protein-coupled receptor kinase 2 (GRK2), which phosphorylates serine/threonine residues on CXCR2 (a G protein-coupled receptor expressed on the neutrophil surface) [39], resulting in recycling or degradation of neutrophils [40]. This reduces the rolling, adhesion, and migration of neutrophils to the infection site [41], resulting in impaired clearance of infection and may explain the higher incidence of metastatic complications in KPPLA.

Our study also demonstrated lower mortality in KPPLA compared to N-KPPLA. This finding was similarly described in narrative reviews and various studies [42,43,44,45]. There are several factors which we need to consider. Firstly, baseline demographics and illness severity may predispose N-KPPLA to worse outcomes. For instance, Chen et al. in 2007, who studied 202 patients, showed higher, but not statistically significant mortality in ECPLA compared to KPPLA (26% vs. 4%, adjusted OR 4.2, *p* = 0.105) [16]. Multivariate analysis on predictors of mortality in ECPLA showed that Acute Physiology and Chronic Health Evaluation (APACHE) II score and underlying malignancy were predictors of mortality (adjusted OR 1.7, 95% CI: 1.1–2.6, *p* = 0.021, and adjusted OR 26 (95% CI: 1.8–370, *p* = 0.016), respectively) [16]. None of the individual studies showed significantly lower mortality in KPPLA than N-KPPLA. However, overall mortality across all studies is about half in KPPLA compared to N-KPPLA (KPPLA: 3.9% vs. N-KPPLA 7.6%). Pooled OR in our meta-analysis also showed significantly lower mortality (OR 0.51, 95% CI: 0.34, 0.78, *p* < 0.001). Repetitive testing of significance by pooling of results in the meta-analysis may have resulted in the rejection of null hypothesis and type 1 error (i.e., demonstrating that there is statistically significant lower mortality in KPPLA even though the results may not be significant). Therefore, cumulative analysis and TSA was performed to control for random errors. Both methods showed that no further studies are required to demonstrate lower mortality in KPPLA, and the rejection of the null hypothesis is not by chance. However, it cannot be determined if the lower mortality is due to fewer co-morbidities, disease attributes, or due to differences in virulent factors. 

Interestingly, while it was shown that overall metastatic complications are higher in KPPLA, KPPLA is associated with lower mortality. Plausible explanations for higher overall metastatic complications have been described above: virulence factors and host factors (i.e., DM causing impaired immune function). Impaired immune function secondary to DM has resulted in higher mortality [41]. This was not observed in our study. The management of sepsis, particularly PLA involves a multi-modal approach with an interdisciplinary team involving surgeons, interventional radiologists, infectious disease physicians, and nurses [8]. Apart from treatment with antibiotics, source control is crucial [46]. We showed that KPPLA is associated with solitary and unilobar abscesses, which are more easily drained. This is reinforced by our findings that more KPPLA were likely to undergo PD (*p* = 0.01) and had a trend towards lower incidence of SD (*p* = 0.11), though this did not reach statistical significance. SD is usually reserved for PLA refractory to less invasive therapy (i.e., antibiotics, percutaneous aspiration, and/or drainage) [9]. We also found that KPPLA is associated with multi-loculation. Some series have shown that multi-loculation results in the compartmentalization of abscesses and may lead to poor drainage, requiring the need for SD for a breakdown of loculations [47,48]. Multivariate analysis by Haider et al. demonstrated that the presence of multiple abscesses is independently associated with primary failure of PD, but not multi-loculation [49]. This may explain why despite the association of KPPLA with multi-loculation, there is adequate drainage with lower mortality.

An interesting consideration is that, while we showed KPPLA has lower mortality compared to N-KPPLA, KP is not associated with lower mortality in other pathologies. For instance, Chan et al. demonstrated that KP bacteremia is associated with higher 30-day mortality (OR 6.09, 95% CI: 1.27–29.10, *p* = 0.025) compared to *Escherichia coli* in acute cholangitis [50]. They postulated that higher mortality in KP bacteremia might be due to virulence factors and higher antibiotic resistance in KP [51]. A plausible explanation for the difference in findings between pathologies with the same organism, i.e., KP, may be due to demographic and radiological differences. For instance, KPPLA is more common in younger patients and in patients without underlying hepatobiliary disease or malignancy. Malignancy is a known predictor of poor prognosis in several infections; haematological malignancies result in dysfunctional white blood cells, which reduces immunological function. Patients may also be on cytotoxic chemotherapy for solid organ malignancies, resulting in an immunocompromised state [52,53]. PLA of biliary origin has also been shown to be independently associated with mortality [54]. Underlying hepatobiliary diseases may result in co-existing biliary tract infections, resulting in worse outcomes [16]. While DM results in an immunocompromised state resulting in an increased risk of infections [55], it is also more often associated with cryptogenic PLA, younger patients, and better outcomes [16]. As mentioned above, there is increasing antibiotic resistance in KP. A recent study on 370 isolates of KP showed detection of NDM-1, OXA-48, and ESBL (TEM, SHV, and CTX-M) in two K2 serotypes of KP [56]. While our current results show lower mortality in KPPLA, this may change in the future with multi-drug resistant strains.

Gas formation is an important radiological finding in PLA. Reports have associated the presence of gas formation, i.e., gas-forming PLA (GFPLA) with KP and higher mortality [7,57,58]. A more recent study in 2020, however showed similar mortality and incidence of KP between GFPLA and non-GFPLA [59]. Our meta-analysis failed to demonstrate significant differences in GFPLA between KPPLA and N-KPPLA (OR 1.04, 95% CI: 0.59, 1.83, *p* = 0.90). Gas formation is an imaging biomarker that is due to the fermentation of mixed acid within the abscess by formic hydrogenlyase, an enzyme produced by certain bacteria [60]. A narrative review in 2018 by Thng et al. on GFPLA showed that KP was more common in GFPLA compared to non-GFPLA in various studies; for instance, Chou et al. studied 424 patients and showed 87.0% of patients with KPPLA had gas formation compared to 63.2% without gas formation [61]. However, our study cannot confirm the clinical outcomes between GFPLA and non-GFPLA. 

Our study has its strengths. Firstly, this is the first systematic review and meta-analysis to review the literature of KPPLA versus N-KPPLA. We also obtained a large overall sample size with 16 included articles and 5127 patients. Our study also utilized cumulative analysis and TSA to control for random error and concluded that no further studies are required to investigate mortality differences between KPPLA and N-KPPLA. However, our study also has limitations. Firstly, non-KP bacteria were grouped as “N-KPPLA”. No subgroup analysis was performed for mortality in patients with metastatic complications versus no metastatic complications due to the low number of studies reporting these outcomes. We also did not analyze the virulence of KPPLA and non-KPPLA in the study (e.g., whether worse outcomes are due to K1 or K2 capsular serotypes) as these were not included in the studies which compared outcomes of KPPLA versus non-KPPLA apart from one study [18]. This also highlights the lack of reporting on microbiology details alongside clinical outcomes and vice versa, and calls for multidisciplinary collaboration. KPPLA has also been associated with the risk of subsequent colorectal cancer; however, this was only reported in one study, and hence we were unable to perform a quantitative analysis on the risk of colorectal cancer [23]. While we collected data on underlying hepatobiliary diseases and history of malignancy, the majority of included studies did not specify the details, e.g., type of malignancy (hematological vs. solid organ) or whether the patient is on chemotherapy, and these may confound outcomes. Lastly, the impact of local resource constraints, compliance to sepsis bundle, implementation of antibiotic stewardship initiatives, and lack of antibiotic resistance data limits the generalizability of our results. 

## 4. Materials and Methods

### 4.1. Study Selection and Search Strategy

This systematic review and meta-analysis were performed according to the PRISMA (Preferred Reporting Items for Systematic reviews and Meta-Analyses) guidelines [62]. The study protocol was registered at PROSPERO (Ref no: CRD42022328345). We systematically searched the following databases (PubMed, Embase, The Cochrane Library, and Scopus) for studies published from inception to 14 May 2022. A combination of search terms (“pyogenic liver abscess” OR “pyogenic hepatic abscess”) AND (“*Klebsiella pneumoniae*” OR “*K. pneumoniae*”) was used. The complete search strategy for each database is appended in Supplementary Table S1.

Inclusion studies were randomized controlled trials (RCTs) and non-RCTs (NRCT) comparing the clinical demographics, biochemistry findings, radiological findings, and/or clinical outcomes between KPPLA and N-KPPLA. Exclusion criteria were (a) studies that were not relevant to PLA, (b) studies without a comparator group (i.e., studies which evaluated only KPPLA or N-KPPLA), (c) basic science studies investigating the prevalence or expression of genes or virulence factors without direct clinical relevance (e.g., description of clinical demographics or outcomes), and (d) type of articles (conference abstracts, studies which have incomplete information, case report or series, editorials, expert opinions, review articles without original data, and non-English studies).

After removing duplicates, abstracts were screened for potential inclusion independently by two authors (K.S.C, C.T.W.C). Subsequently, full texts of included studies were reviewed and selected based on the inclusion and exclusion criteria. There were no studies reporting data on the same cohort of patients. Any discrepancies were resolved by consensus and review by the senior author (V.G.S).

### 4.2. Data Extraction

Data extraction was independently conducted by two authors (K.S.C, C.T.W.C). The following variables were extracted from each study: publication details (name of first author, publication year, and country), study characteristics (sample size, sex, age, sex, co-morbidities (DM, history of biliary diseases or malignancy), presenting symptoms (fever or abdominal pain), biochemistry values (total white cell count, bilirubin level, C-reactive protein level), radiological findings (size, number and location of the abscess, and presence of gas or multiloculated abscess on ultrasound (US) and/or computed tomography (CT) scan). Study outcomes were the type of treatment received (conservative with antibiotics and without any drainage, need for percutaneous aspiration or PD, surgical drainage (SD)), total duration of antibiotic, presence of metastatic infection, septic shock, need for intensive care unit (ICU) admission, length of stay (LOS), recurrence and mortality. A metastatic complication was defined as bacteremia resulting in distant complications secondary to primary PLA. Distant complications were defined as endophthalmitis, intra-cranial abscess/meningitis, or thrombophlebitis of hepatic veins and their tributaries and/or inferior vena cava. Overall metastatic infection was defined as the presence of distant complications; for studies that reported any of the complications. Mortality was defined as either 30-day, 6-week (one study included 6-week mortality [24]), or in-hospital mortality.

### 4.3. Assessment of Study Quality 

Two independent authors performed a quality assessment of the finalized study (K.S.C, C.T.W.C). NRCTs were assessed using the modified Newcastle-Ottawa scale (Supplementary Table S2) [63]. Disagreements between authors were resolved by discussion with the senior author (V.G.S).

### 4.4. Statistical Analysis

Study variables were extracted to Microsoft^®^ Excel 365 (Microsoft^®^, Washington, WA, USA). Categorical variables were described as n (%), and continuous variables were described as mean ± standard deviation or median (interquartile range) unless otherwise specified. For studies that only expressed median and range or interquartile range, mean and standard deviation were estimated using methods described by Wan et al. for quantitative analysis [64]. Meta-analysis was performed using Stata (version 17.0, StataCorp, College Station, TX, USA). Outcomes with dichotomous outcomes were expressed as odds ratios (ORs) with a 95% confidence interval (CI), which were calculated using the Mantel-Haenszel method. Outcomes with continuous outcomes were expressed as mean differences (MD) with 95% CI, pooled with the inverse-variance method. Statistical significance was defined as *p* < 0.05. Heterogeneity was assessed using Cochrane’s Q and quantified by I^2^. Heterogeneity was defined by I^2^ > 50%. A fixed-effects model was used when I^2^ ≤ 50%, and a random-effects model using restricted maximum likelihood (REML) was used when I^2^ > 50%. Funnel plot and Egger’s regression test was used to test for publication bias [65].

Cumulative analysis was also performed using Stata to determine temporal trends in PLA, given improvement in multi-modal care of PLA and mortality in recent years [2,66]. Repetitive testing of included studies in meta-analysis accrues data, and repeated testing of significance can lead to rejection of the null hypothesis [67]. Bias from included studies with low quality and small sample sizes may also result in spurious *p*-values [68]. Trial sequential analysis (TSA) allows for controlling for random errors and assessing whether further trials are required to be conducted [69]. The Trial Sequential Analysis software (The Copenhagen Trial Unit, Copenhagen, Denmark) was used for TSA for mortality outcomes [70]. A TSA model was produced assuming a 5% (two-sided α = 0.05) Type 1 error and 80% statistical power based on the O’Brien–Fleming alpha-spending function. The required information size adjusted for this meta-analysis was calculated to determine if the evidence was reliable [68]. The cumulative Z-curve which represents the cumulative Z values from accumulating data from addition of individual trials is also plotted. If the cumulative Z-curve crosses the trial sequential monitoring boundary (TSMB), this was determined as achieving intervention effect with firm evidence. The TSMBs are analogous to interim monitoring boundaries in a single trial and adjust the *p*-value required to reach statistical significance according to the number of participants and events in a meta-analysis; a lower number of participants and events results in more restrictive monitoring boundaries and lowers the *p*-value required to obtain statistical significance [71].

## 5. Conclusions

This systematic review and meta-analysis comparing clinical outcomes of KPPLA vs. N-KPPLA demonstrated that KPPLA is associated with higher overall metastatic complications but lower mortality. Lower mortality may be due to patients being younger and without underlying hepatobiliary disease or malignancy, and abscess characteristics (solitary and unilobar) permitting easier drainage. Future studies may consider reporting other clinical outcomes such as duration of antibiotic use, recurrence, failure of initial therapy, and re-admission rates.

## Figures and Tables

**Table 1:** Study characteristics of the included 4 randomized controlled trials and 25 non-randomized controlled trials.

No	Author, Year	Type of Study	Country	Study Period	Definition of KPPLA	Definition of N-KPPLA	No. of Patients (KPPLA/N-KPPLA)	Age (KPPLA/N-KPPLA)	Males, n (KPPLA/N-KPPLA)	Diabetes Mellitus, n (%) (KPPLA/N-KPPLA)	History of Malignancy, n (%) (KPPLA/N-KPPLA)	Pus Culture Done, n (%)	Blood Culture Done, n (%)
1	Kong, 2017 [17]	Retrospective	China	Jan 2010–Dec 2014	Monomicrobial	Polymicrobial (which may include KP), or non-KP monomicrobial	217 (165/52)	55.8 ± 11.6/56.1 ± 11.2	106/30	71/11	NR	NR	NR
2	Yin, 2021 [14]	Retrospective	China	Jan 2006–Dec 2017	Monomicrobial	Monomicrobial *EC*	606 (576/30)	NR		275/8	2/1	KPPLA: 576/576 N-KPPLA: 30/30	NR
3	Chen, 2007 [16]	Retrospective	Taiwan	Jul 2000 –June 2005	Monomicrobial and polymicrobial (n = 11/147)	Polymicrobial (excluding KP), or monomicrobial	202 (147/55)	NR	101/27	82/19	15/18	KPPLA: 147/147 N-KPPLA: 55/55	KPPLA: 81/147 N-KPPLA: 19/55
4	Wang, 2013 [18]	Retrospective	China	Jan 2008–Dec 2011	Monomicrobial	Polymicrobial (which may include KP), or non-KP monomicrobial	131 (101/30)	53.93 ± 1.45/56.33 ± 2.25	77/6	59/7	NR	KPPLA: 131/131 N-KPPLA: 300/30	NR
5	Kim, 2007 [19]	Retrospective	Korea	Jan 2000–Oct 2005	Monomicrobial and polymicrobial (n = 5/58)	Polymicrobial (n = 15/56) (excluding KP), or monomicrobial	114 (58/56)	59.8 ± 13.6/61.5 ± 14.8	31/31	17/2	3/0	NR	NR
6	Alsaif, 2011 [13]	Retrospective	Singapore	Jul 2003 –Jul 2010	Monomicrobial	Polymicrobial (which may include KP n = 7/39), or non-KP monomicrobial	131 (92/39)	56.5 ± 15.8/59.7 ± 12.4	67/27	45/22	9/4	KPPLA: 84/92 N-KPPLA: 35/39	KPPLA: 49/92 N-KPPLA: 18/39
7	Lee, 2011 [20]	Retrospective	Korea	Dec 2001–Dec 2006	Monomicrobial	Polymicrobial (which may include KP n = 12/70), or non-KP monomicrobial	129 (59/70)	62.2 (range 36–86)/65.8 (range 25–91)	33/32	36/36	NR	NR	NR
8	Li, 2010 [21]	Retrospective	China	Mar 2001–Sep 2009	Monomicrobial	Monomicrobial non-KP	162 (106/56)	58.02 ± 2.4/56.04 ± 3.52	77/39	57/14	3/7	NR	NR
9	Baek, 2017 [22]	Retrospective	Korea	Mar 2011–Mar 2015	Monomicrobial	Polymicrobial (which may include KP), or non-KP monomicrobial	87 (60/27)	62.3 ± 13.5/64.5 ± 11.5	38/17	15/5	4/13	KPPLA: 37/60 N-KPPLA: 13/27	KPPLA: 3/60 N-KPPLA: 1/27
10	Huang, 2012 [23]	Retrospective	Taiwan	Jan 2000–Dec 2009	Monomicrobial and polymicrobial	Polymicrobial (excluding KP), or monomicrobial	2294 (1194/1100)	56.6 ± 13.2/59.3 ± 15.4	810/696	661/323	51/252	NR	NR
11	Chen, 2014 [24]	Retrospective	Taiwan	Jan 2009–Dec 2009	Monomicrobial	Polymicrobial (n = 2/39) (including KP), or monomicrobial	134 (95/39)	NR	NR	NR	NR	NR	NR
12	Shelat, 2015 [8]	Retrospective	Singapore	Jan 2003–Dec 2011	Monomicrobial	Monomicrobial *EC* (except for 1 case with both KP and EC)	288 (264/24)	62 (range 25–94)/ 68 (range 21–89)	160/12	101/10	NR	NR	NR
13	Hui, 2007 [25]	Retrospective	Hong Kong	Jul 1997–June 2002	Monomicrobial	Polymicrobial (n = 27/30) (including KP n = 11/27), or monomicrobial	80 (50/30)	64.4 ± 16.7/69.3 ± 12	26/17	28/6	3/8	NR	KPPLA: 29/50 N-KPPLA: 15/33
14	Lee, 2020 [26]	Retrospective	Korea	Jan 2010–May 2018	Monomicrobial	Polymicrobial (which may include KP), or non-KP monomicrobial	219 (133/86)	57.15 ± 8.25/62.87 ± 5.11	91/61	57/17	13/14	KPPLA: 68/133 N-KPPLA: 53/86	KPPLA: 20/133 N-KPPLA: 13/86
15	Qian, 2016 [27]	Retrospective	China	May 1994–Dec 2015	Monomicrobial and polymicrobial (n = 3)	Polymicrobial (excluding KP), or monomicrobial	296 (189/107)	59.5 ± 13.1/58.4 ± 12.1	115/66	94/39	15/30	KPPLA: 163/167 N-KPPLA: 82/91	KPPLA: 51/153 N-KPPLA: 42/75
16	Mousa, 2021 [28]	Retrospective	United Arab Emirates	Jan 2012–Dec 2018	NR	NR	37 (16/21)	NR	12/16	7/5	1/2	KPPLA: 16/16 N-KPPLA: 17/21	KPPLA: 5/16 N-KPPLA: 3/21

All categorical variables are expressed as n (%) unless otherwise specified. All continuous variables are expressed as mean ± standard deviation unless otherwise specified. EC: Escherichia coli; KP: Klebsiella pneumoniae; KPPLA: Klebsiella pneumoniae pyogenic liver abscess; N-KPPLA: non-Klebsiella pneumoniae pyogenic liver abscess NR: Not reported.

**Table 2:** Radiological findings and outcomes of *Klebsiella pneumoniae* pyogenic liver abscess (KPPLA) vs. non-KPPLA (N-KPPLA).

No	First Author, Year	Radiological Findings				Exclusion of Conservatively Managed Only	Conservative Management Only, n (Yes/Total)	Surgical Drainage, n (Yes/Total)	Length of Stay (LOS), Days (KPPLA/N-KPPLA)	Any Metastatic Complications, n (Yes/Total)	ENDOPHTHALMITIS, n (Yes/Total)	Mortality #, n (Yes/Total)
		Imaging Modality	Size, cm (KPPLA/N-KPPLA)	Multiple Abscess, n (KPPLA/N-KPPLA)	Gas Formation, n (KPPLA/N-KPPLA)							
1	Kong, 2017 [17]	US/CT	NR	NR	NR	No	KPPLA: 0/165 N-KPPLA: 0/52	KPPLA: 18/165 N-KPPLA: 6/52	NR	NR	NR	KPPLA: 4/165 N-KPPLA: 0/52
2	Yin, 2021 [14]	US/CT	NR	120/6	53/1	No	KPPLA: 35/576 N-KPPLA: 6/30	KPPLA: 2/576 N-KPPLA: 1/30	NR	NR	KPPLA: 9/576 N-KPPLA: 1/30	KPPLA: 6/576 N-KPPLA: 0/30
3	Chen, 2007 [16]	US/CT	>5 cm: KPPLA: 104/147 N-KPPLA: 32/55	32/37	36/9	No	KPPLA: 6/147 N-KPPLA: 6/55	KPPLA: 3/147 N-KPPLA: 0/55	NR	KPPLA: 20/147 N-KPPLA: 5/55	KPPLA: 5/147 N-KPPLA: 0/55	KPPLA: 6/147 N-KPPLA: 14/55
4	Wang, 2013 [18]	US	6.34 ± 0.37/7.30 ± 0.27	17/4	35/4	NR	NR	NR	NR	NR	NR	NR
5	Kim, 2007 [19]	CT	≥5 cm: KPPLA: 46/58 N-KPPLA: 27/56	20/23	9/5	NR	NR	NR	NR	KPPLA: 3/58 N-KPPLA: 0/56	KPPLA: 0/58 N-KPPLA: 0/56	NR
6	Alsaif, 2011 [13]	CT	7.3 ± 2.8/ 7.8 ± 2.8	19/17	11/6	Yes	KPPLA: 0/92 N-KPPLA: 0/39	KPPLA: 8/92 N-KPPLA: 6/39	14 (range 2–56)/16 (range 7–72)	NR	NR	KPPLA: 3/92 N-KPPLA: 4/39
7	Lee, 2011 [20]	CT	NR	NR	52/64	No	KPPLA: 0/59 N-KPPLA: 0/70	NR	NR	KPPLA: 12/59 N-KPPLA: 1/70	KPPLA: 5/59 N-KPPLA: 0/70	NR
8	Li, 2010 [21]	US	7.39 ± 0.24/7.38 ± 0.32	13/7	27/6	No	KPPLA: 0/106 N-KPPLA: NR	KPPLA: 0/106 N-KPPLA: NR	NR	NR	NR	KPPLA: 1/106 N-KPPLA: 0/56
9	Baek, 2017 [22]	CT	6.9 ± 2.2/ 6.4 ± 2.3	11/9	5/7	Yes	KPPLA: 0/60 N-KPPLA: 0/27	KPPLA: 0/60 N-KPPLA: 0/27	23.45 ± 9.7/27.86 ± 16.52	NR	NR	NR
10	Huang, 2012 [23]	NR	NR	169/282	NR	NR	NR	NR	NR	NR	NR	NR
11	Chen, 2014 [24]	US	NR	2/5	21/7	NR	NR	NR	NR	NR	NR	6-week mortality: KPPLA: 2/95 N-KPPLA: 4/39
12	Shelat, 2015 [8]	CT	6 (range 0.8–15.4)/4 (range 1–11.2)	79/7	NR	No	KPPLA: 95/264 N-KPPLA: 14/24	KPPLA: 0/264 N-KPPLA: 0/24	14 (range 1–80)/14 (range 4–99)	NR	NR	KPPLA: 27/264 N-KPPLA: 4/24
13	Hui, 2007 [25]	US	6.98 ± 2.6/ 8.6 ± 8.4	7/9	0/1	NR	NR	NR	27.3 ± 19.1/22.2 ± 13.6	KPPLA: 11/50 N-KPPLA: 3/33	NR	KPPLA: 7/50 N-KPPLA: 6/33
14	Lee, 2020 [26]	CT	7.1 ± 3.5/7.9 ± 1.9	41/52	15/17	Yes	KPPLA: 0/133 N-KPPLA: 0/86	KPPLA: 8/133 N-KPPLA: 8/86	12 (range 3–36)/19 (range 5–59)	KPPLA: 23/133 N-KPPLA: 7/86	KPPLA: 1/133 N-KPPLA: 0/86	KPPLA: 5/133 N-KPPLA: 3/86
15	Qian, 2016 [27]	NR	7.1 ± 2.5/ 6.5 ± 2.7	38/23	NR	No	KPPLA: 23/189 N-KPPLA: 17/107	KPPLA: 16/189 N-KPPLA: 15/107	18.7 ± 13.1/18.5 ± 12.5	KPPLA: 20/189 N-KPPLA: 4/107	KPPLA: 0/189 N-KPPLA: NR	KPPLA: 11/189 N-KPPLA: 6/107
16	Mousa, 2021 [28]	NR	NR	NR	NR	No	KPPLA: 0/16 N-KPPLA: 0/21	KPPLA: 0/16 N-KPPLA: 0/21	NR	NR	NR	KPPLA: 0/16 N-KPPLA: 0/21

All categorical variables are expressed as n (%) unless otherwise specified. All continuous variables are expressed as mean ± standard deviation unless otherwise specified. # Mortality is defined as either 30-day mortality or in-hospital mortality as defined in the original studies. CT: Computed tomography; KP: Klebsiella pneumoniae; KPPLA: Klebsiella pneumoniae pyogenic liver abscess; N-KPPLA: non-Klebsiella pneumoniae pyogenic liver abscess. NR: Not reported; US: Ultrasonography.

## Data Availability

Data may be extracted directly from the included articles as these articles are publicly available on research databases.

## Supplementary Materials

The following are available online at https://www.mdpi.com/article/10.3390/pathogens11090976/s1, Supplementary Table S1: Search strategy used in all the databases; Supplementary Table S2: Quality assessment of all included observational studies (n = 16) using the Newcastle-Ottawa Scale; Supplementary Figure S1: Funnel plots of included studies (A) Clinical demographics, (B) Radiological findings and (C) Clinical outcomes; Supplementary Figure S2: Clinical demographics comparing KPPLA versus N-KPPLA: (A) Age, (B) Sex, (C) Diabetes mellitus, (D) History of hepatobiliary disease and (E) History of malignancy. KPPLA: Klebsiella pneumoniae pyogenic liver abscess; N-KPPLA: Non-KPPLA; Supplementary Figure S3: Radiological findings comparing KPPLA versus non-KPPLA: (A) Multiple abscess, (B) Bilobar location, (C) Multi-loculation, (D) Gas formation, (E) Size, and (F) Presence of solid component; Supplementary Figure S4: Clinical outcomes of KPPLA versus N-KPPLA: (A) Length of stay, (B) Overall metastatic infections, (C) Endophthalmitis, (D) Brain abscess/meningitis, and (E) Mortality.
