# Supplementary material for: Demographics, Radiological Findings, and Clinical Outcomes of Klebsiella pneumonia vs. Non-Klebsiella pneumoniae Pyogenic Liver Abscess: A Systematic Review and Meta-Analysis with Trial Sequential Analysis
Source: Pathogens. 2022 Aug 26;11(9):976. doi: 10.3390/pathogens11090976 (PMC9505935; doi:10.3390/pathogens11090976)

Supplementary Material

Supplementary Table S1: Search strategy used in all the databases

Database	Search strategy
PubMed	(Liver Abscess, Pyogenic[MeSH Terms]) OR (Pyogenic Hepatic Abscess*[Title/Abstract]) OR (Pyogenic Hepatic Abscess*[Other Term]) OR (Pyogenic Liver Abscess*[Title/Abstract]) OR (Pyogenic Liver Abscess*[Other Term]) OR (Liver Abscess*[Other Term]) OR (Liver Abscess*[Title/Abstract]) OR (Hepatic Abscess*[Title/Abstract]) OR (Hepatic Abscess*[Other Term]) OR (Liver Abscess[MeSH Terms]) AND (*Klebsiella pneumoniae*[MeSH Terms]) OR (*Klebsiella pneumoniae*[Title/Abstract]) OR (*Klebsiella pneumoniae*[Other Term]) OR (*K.pneumoniae*[Title/Abstract]) OR (*K.pneumoniae*[Other Term]) OR (*K. pneumoniae*[Title/Abstract]) OR (*K. pneumoniae*[Other Term]) OR (*Klebsiella**[Title/Abstract]) OR (*Klebsiella**[Other Term])
Embase (Ovid)	("liver abscess pyogenic" or "pyogenic hepatic abscess*" or "pyogenic liver abscess*" or "liver abscess*" or "hepatic abscess*").ti,ab,tw. AND ("*klebsiella pneumoniae*" or "*k.pneumoniae*" or "*k. pneumoniae*" or *klebsiella**).ti,ab,tw.
Scopus	TITLE-ABS-KEY (“liver abscess pyogenic” or “pyogenic hepatic abscess*” or “pyogenic liver abscess*” or “liver abscess*” or “hepatic abscess*”) AND TITLE-ABS-KEY (“*klebsiella pneumoniae*” or “*k.pneumoniae*” or “*k. pneumoniae*” or *klebsiella**)
Cochrane	(“liver abscess pyogenic” or “pyogenic hepatic abscess*” or “pyogenic liver abscess*” or “liver abscess*” or “hepatic abscess*”):ti,ab,kw. AND (“*klebsiella pneumoniae*” or “*k.pneumoniae*” or “*k. pneumoniae*” or *klebsiella**):ti,ab,kw.

Supplementary Table S2: Quality assessment of all included observational studies (n=16) using the Newcastle-Ottawa Scale

No	First author, year	Selection				Comparability	Outcome			Quality score*
		Representativeness of cohort	Selection of non-exposed cohort	Ascertainment of exposure	Demonstration that current outcome was not present at start of study	Comparability of cohorts on basis of study design or analysis	Assessment of outcome	Length of follow-up	Adequacy of follow-up	
1	Kong, 2017	1	1	1	0	0	1	1	1	6
2	Yin, 2021	1	1	1	0	0	1	1	1	6
3	Chen, 2007	1	1	1	0	2	1	1	1	8
4	Wang, 2013	1	1	1	0	0	1	1	1	6
5	Kim, 2007	1	1	1	0	0	1	1	1	6
6	Alsaif, 2011	1	1	1	0	0	1	1	1	6
7	Lee, 2011	1	1	1	0	2	1	1	1	8
8	Li, 2010	1	1	1	0	0	1	1	1	6
9	Baek, 2017	1	1	1	0	2	1	1	1	8
10	Huang, 2012	1	1	1	1	2	1	1	1	9
11	Chen, 2014	1	1	1	0	0	1	1	1	6
12	Shelat, 2015	1	1	1	0	0	1	1	1	6
13	Hui, 2007	1	1	1	0	0	1	1	1	6
14	Lee, 2020	1	1	1	0	2	1	1	1	8
15	Qian, 2016	1	1	1	0	0	1	1	1	6
16	Mousa, 2021	1	1	1	0	1	1	1	1	7

*Quality score of <3: Low quality of evidence, 3-6: Moderate quality of evidence, ⩾7: High quality of evidence

Supplementary Figures

Supplementary Figure S1: Funnel plots of included studies (A) Clinical demographics, (B) Radiological findings and (C) Clinical outcomes

(A) Clinical demographics

Age	Sex
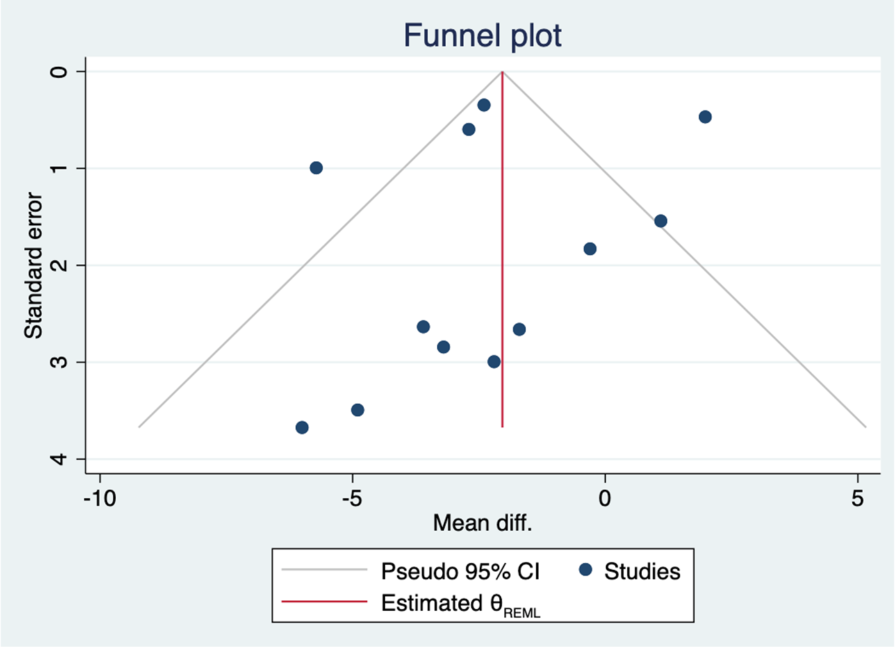	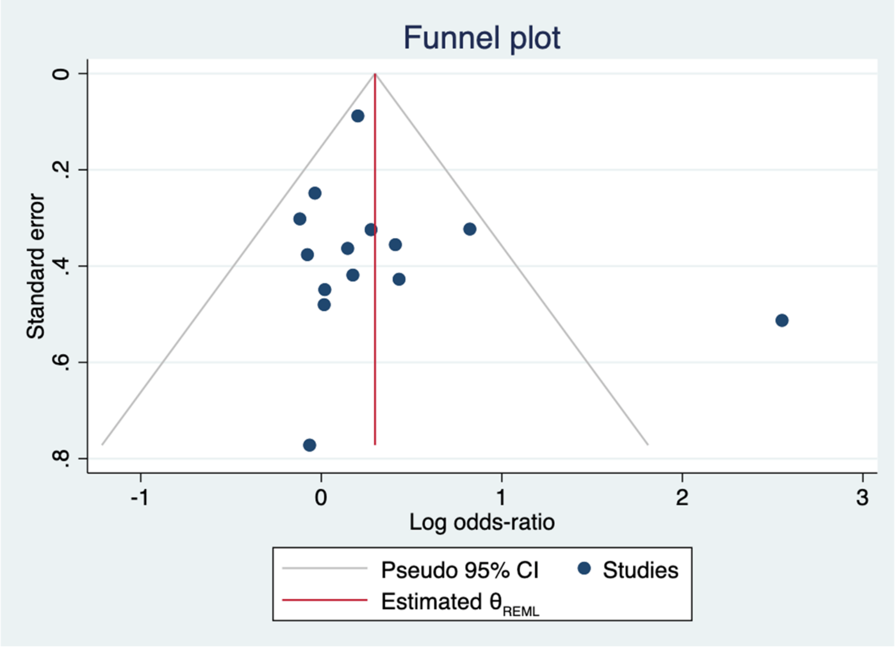

Diabetes mellitus

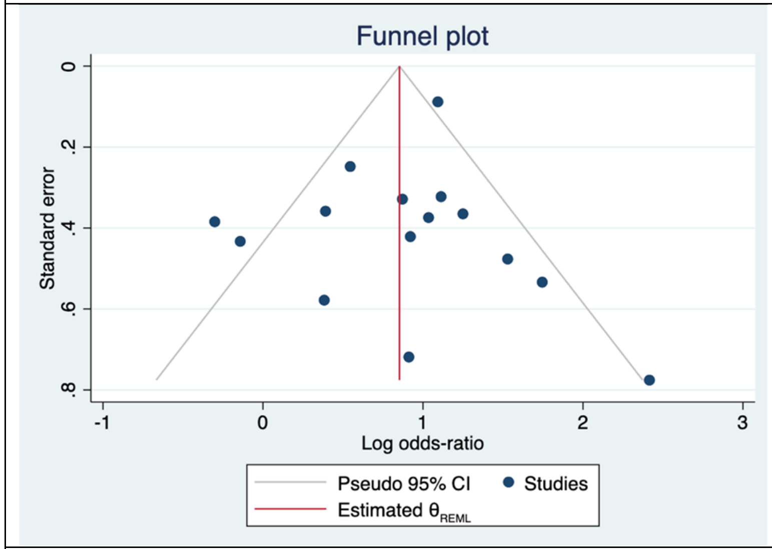

Underlying hepatobiliary disease

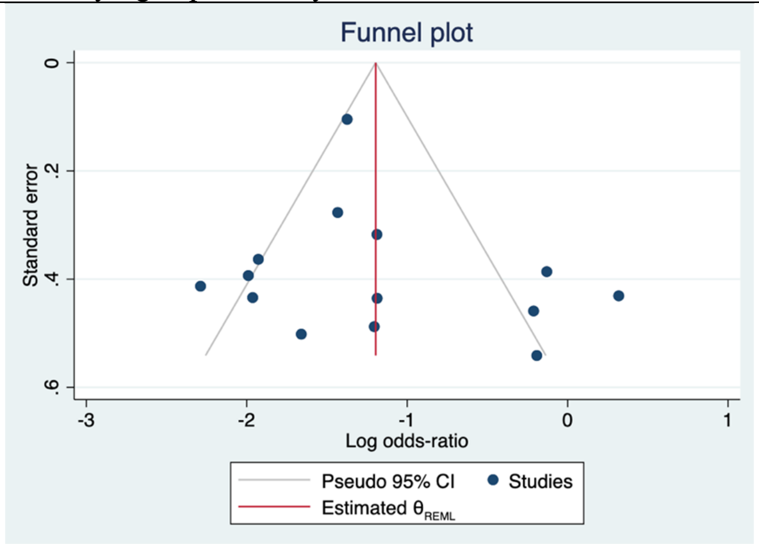

Underlying malignancy

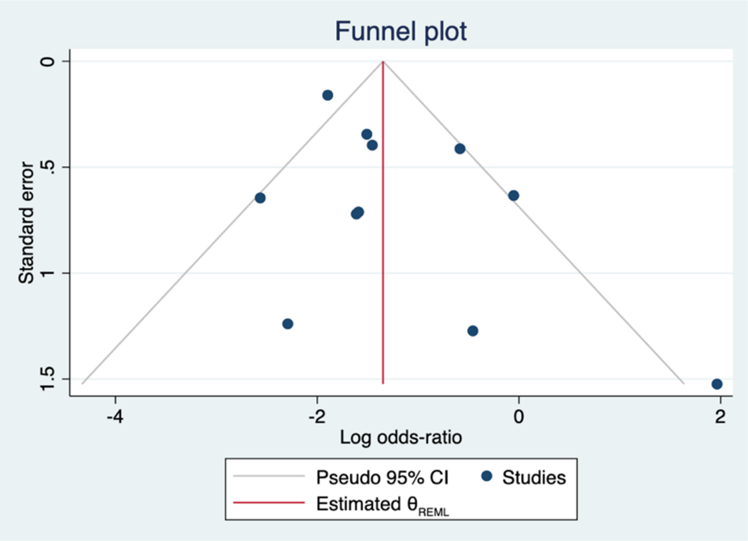

(B) Radiological findings

Size	Multiple abscess
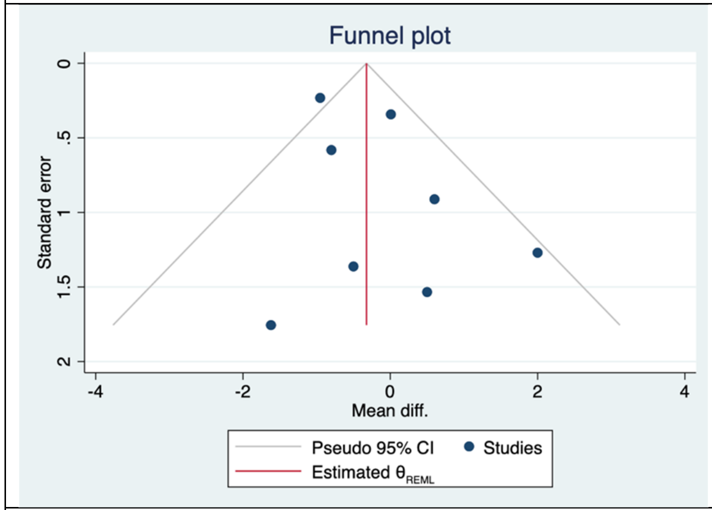	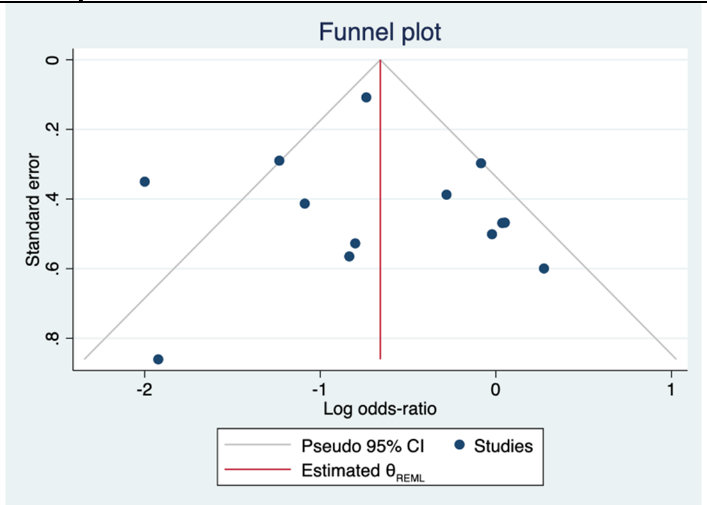
**Multi-loculation**	**Gas formation**
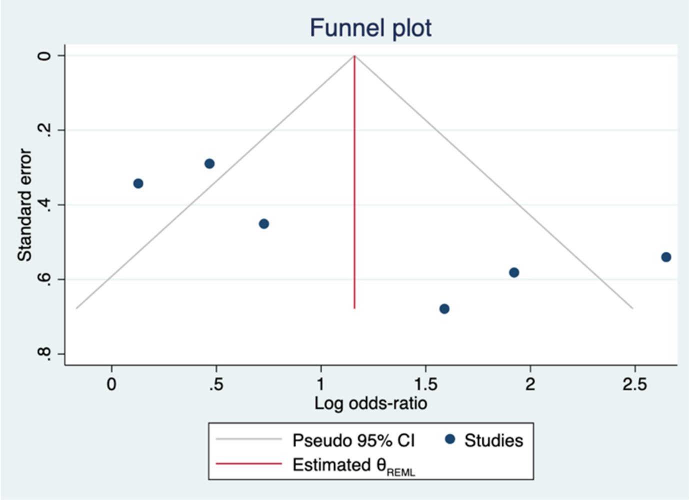	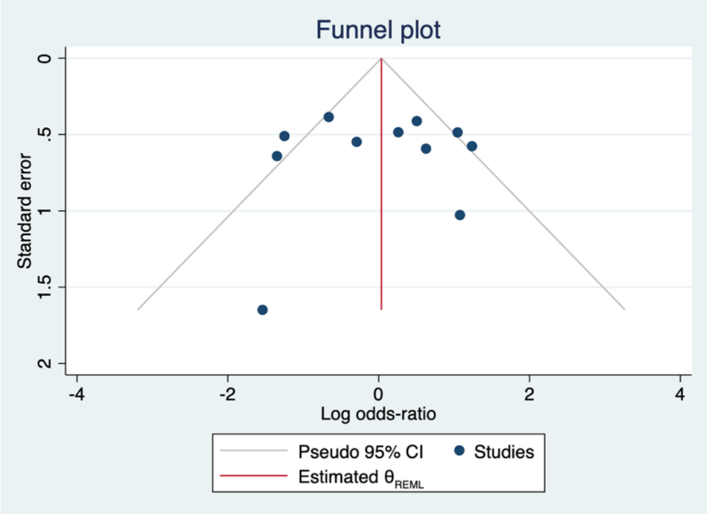

Bilobar	Presence of solid component
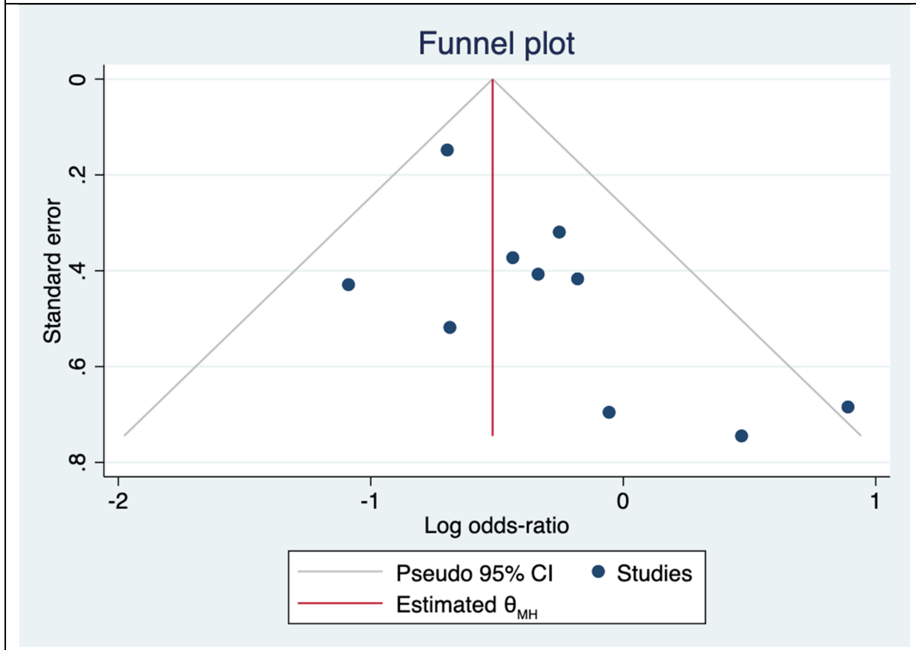	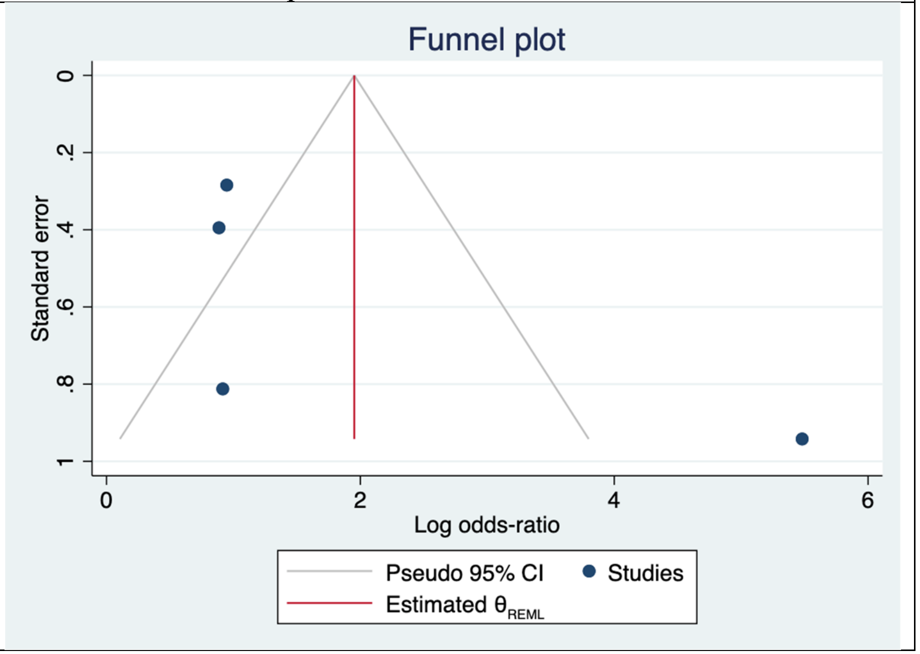

(C) Clinical outcomes

Overall metastatic complications

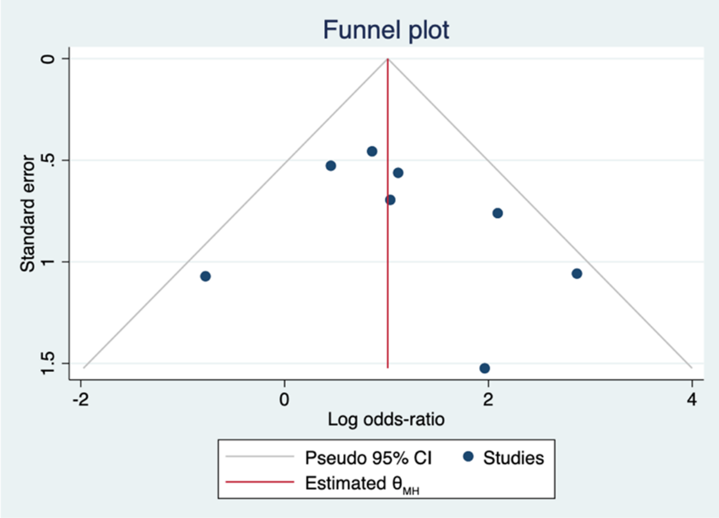

Endophthalmitis

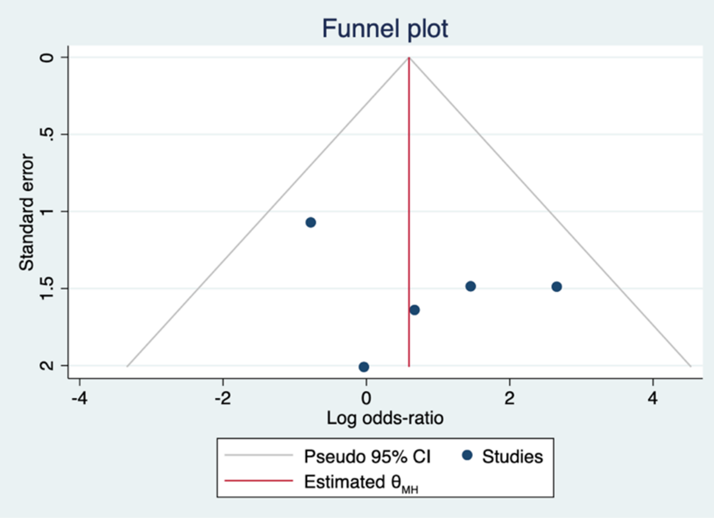

Brain abscess/meningitis

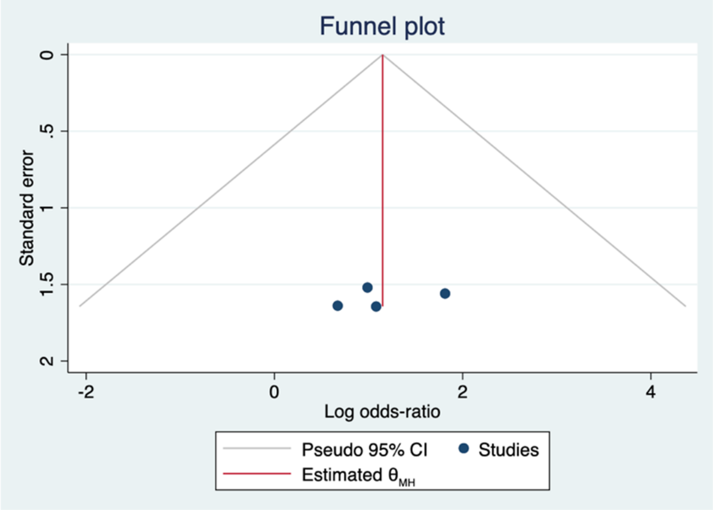

Length of stay

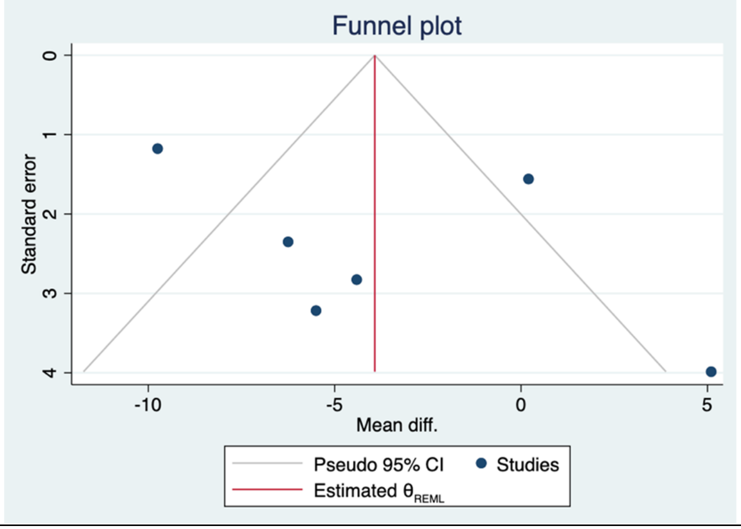

Mortality	
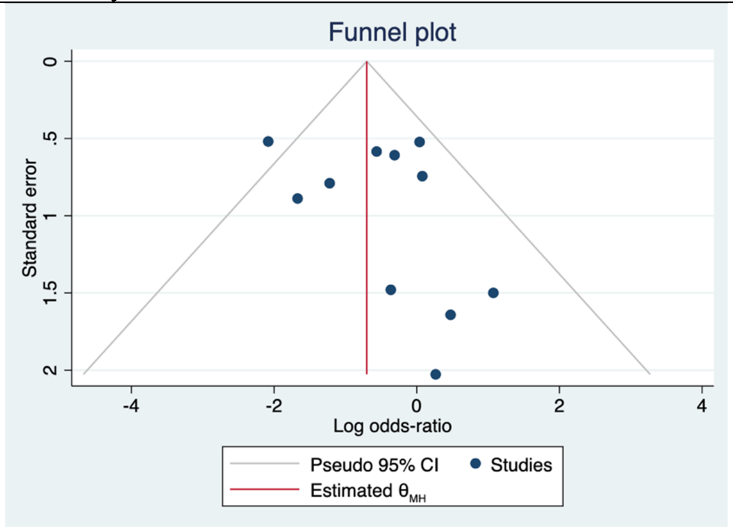Funnel plot<br>Standard error<br>0<br>.5<br>1<br>1.5<br>2<br>-4<br>-2<br>0<br>2<br>4<br>Log odds-ratio<br>Pseudo 95% CI<br>Studies<br>Estimated θMH	

Supplementary Figure S2: Clinical demographics comparing KPPLA versus N-KPPLA: (A) Age, (B) Sex, (C) Diabetes mellitus, (D) History of hepatobiliary disease and (E) History of malignancy. KPPLA: Klebsiella pneumoniae pyogenic liver abscess; N-KPPLA: Non-KPPLA

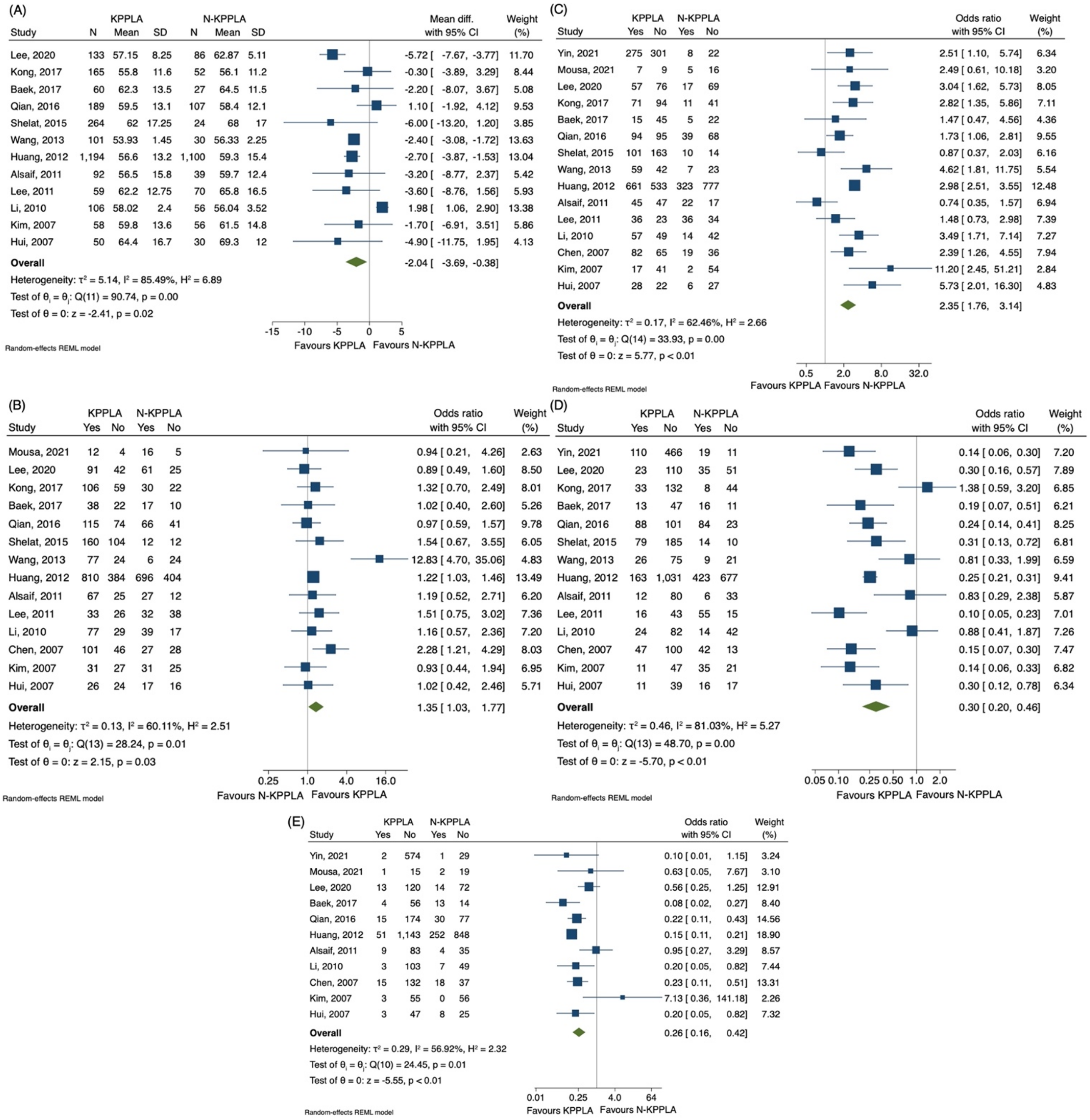

Supplementary Figure S3: Radiological findings comparing KPPLA versus non-KPPLA: (A) Multiple abscess, (B) Bilobar location, (C) Multi-loculation, (D) Gas formation, (E) Size, and (F) Presence of solid component. KPPLA: Klebsiella pneumoniae pyogenic liver abscess; N-KPPLA: Non-KPPLA

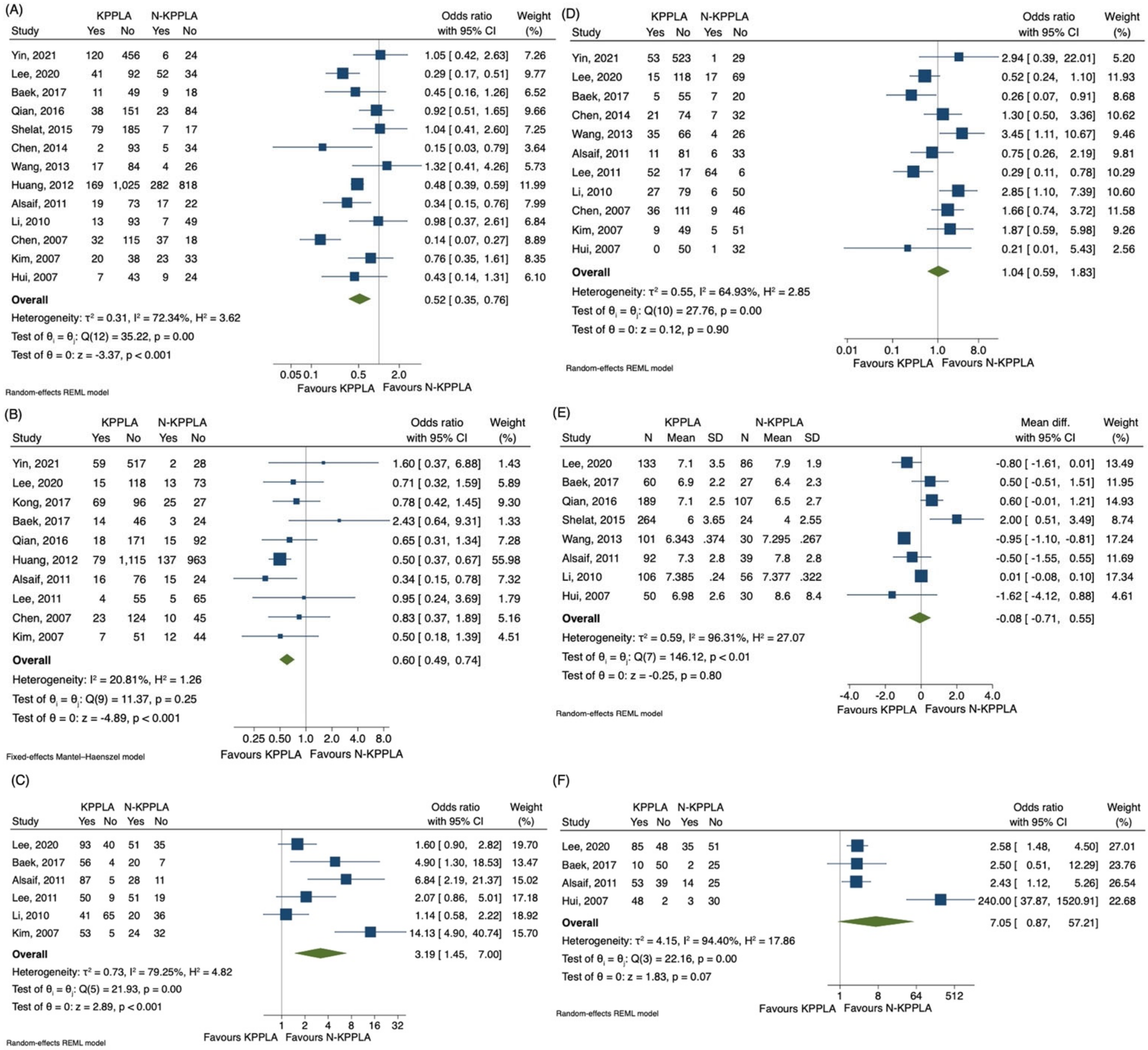

Supplementary Figure S4: Clinical outcomes of KPPLA versus N-KPPLA: (A) Length of stay, (B) Overall metastatic infections, (C) Endophthalmitis, (D) Brain abscess/meningitis, and (E) Mortality. KPPLA: Klebsiella pneumoniae pyogenic liver abscess; N-KPPLA: Non-KPPLA

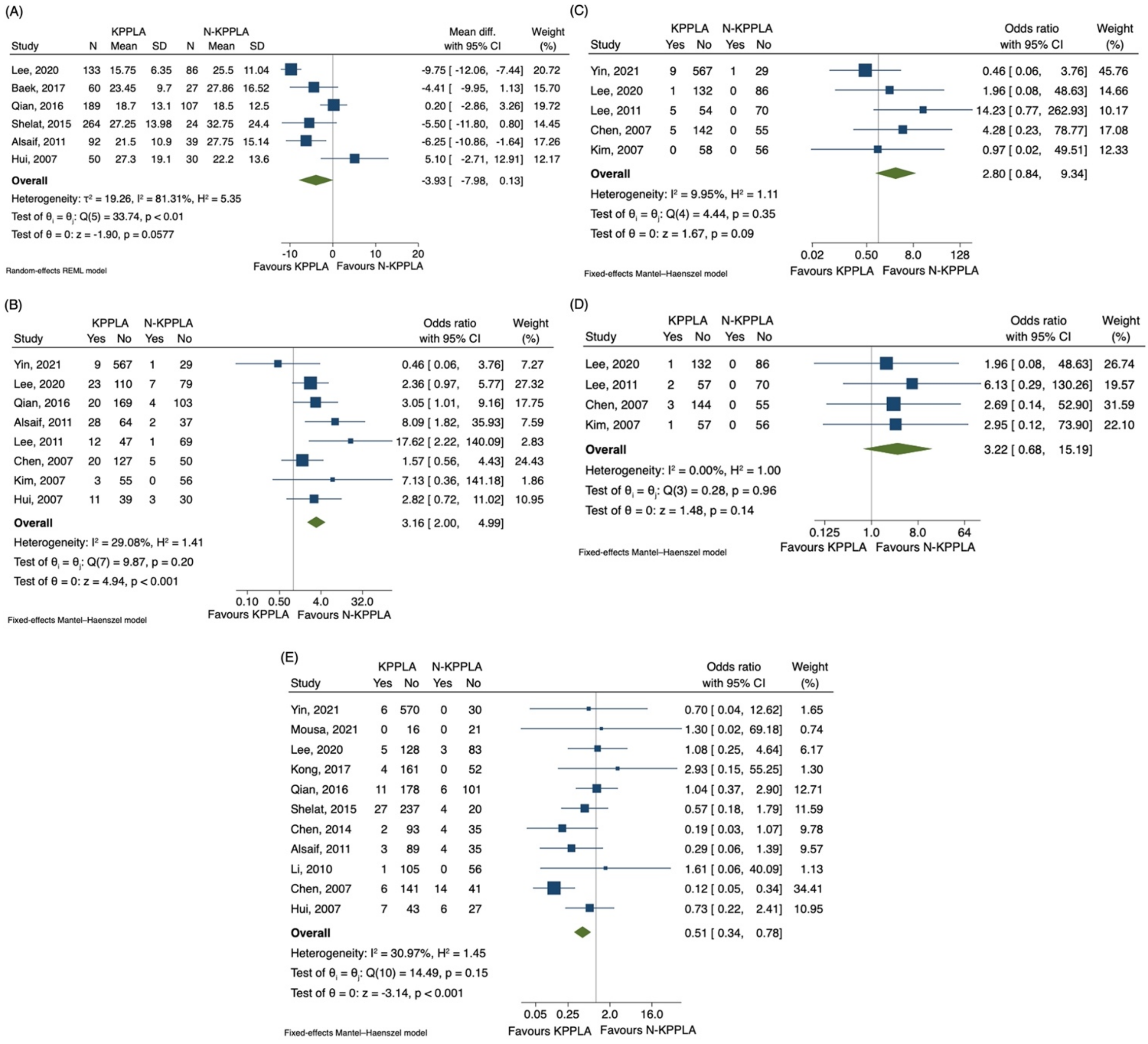

Supplement: Supplementary file 1 [file pathogens-11-00976-s001.zip › pathogens-1856773-supplementary.pdf]
